# The Use of Local Anesthetics in Operations on the Chest and Abdomen1Read at the first meeting of the Eighth District Branch of Medical Society of the State of New York held at Buffalo, August 18 and 19, 1906.

**Published:** 1906-10

**Authors:** Marshall Clinton

**Affiliations:** Buffalo, N.Y.; Lecturer in Surgical Pathology at the University


### BUFFALO MEDICAL JOURNAL.

VOL. LXII. OCTOBER, 1906. No. 3

### ORIGINAL COMMUNICATIONS.

### The Use of Local Anesthetics in Operations on the Chest and Abdomen.[1]

By MARSHALL CLINTON, M. D., Buffalo, N. Y.
Lecturer in Surgical Pathology at the University.

THE great aim of surgery is to practise the art in such a manner as will utilise all the expedients known to science that may have a bearing on the effort to conserve human life. We experiment, try, adapt, or reject various suggestions that are offered for the relief of surgical conditions; reject some because they are technically hard for the surgeon and some because they seem needlessly hard for the patient. Ever since Schleich and his co-workers in the field of infiltration,—or better, pressure anesthesia,—efforts have been made to utilise in selected cases the knowledge they have handed down to us.

In his earlier experience with general anesthetics it was impressed on the mind of the writer that now and then one ran across a patient who had to submit to some relatively simple surgical procedure, who had a needlessly hard time in the earlier days of convalescence, if he was fortunate enough to escape alive. No doubt we see cases of very severe but relatively slowly invading infections where the patient has built up an immunity that is shown in lack of high temperature, rapid pulse and great prostration. In these patients, notably in lesions of the chest and abdomen, rapidly performed slight operations under general anesthetics are followed by an apparent tearing down of the immunity and resistance the patients have so far built up. There seems to be some agent apart from the operative work responsible for the breakdown.

It is not hard to imagine when considering the many factors in the symptom picture of such a case what harm a general anes-

---

1. Read at the first meeting of the Eighth District Branch of Medical Society of the State of New York held at Buffalo, August 18 and 19, 1906.

thetic may do when used on such a patient. If we can utilise some method of local anesthesia that will not produce or be followed by surgical shock, it seems logical to believe that a greater proportion of these patients will recover than when operated under full surgical anesthesia. I say "full surgical anesthesia" advisedly, because it is the term employed in many textbooks to denote the stage of anesthesia arrived at by the use of chloroform or ether. We know, however, that in full surgical anesthesia pain is absent during that period, but that impulses which produce profound surgical shock are transmitted when the patients are under chloroform or ether.

A perfect system of nerve blocking must be devised before we can eliminate surgical shock as a necessary evil in our work. Fortunately, we have several men who are constantly striving to perfect their technic in methods usually followed by marked depression and their later statistics will undoubtedly compare better, case for case, with others in which less painstaking methods are employed. The writer does not feel as sanguine as some advocates of local anesthesia that this procedure will ultimately supplant all others, because so many cases seem unsuited to its employment in that general anethesia offers slight risks in competent hands as well as gives the sweet oblivion that the average not too sick patient wishes. In the use of local anesthesia on the chest and abdomen of course we do not include minor surgery as that should always be done under the use of local anesthetics.

The first series of cases in which it was deemed advisable to use local anesthesia was on patients who had a massive empyema of the chest with rapid labored respirations, small soft rapid pulse, slight cyanosis and not much temperature. You have all seen them; and seen them snuffed out following an anesthetic in a few hours after operation.

My first case was done with a 2% solution of cocaine on a young girl who looked much sicker than her pulse rate and temperature suggested. A line along the skin, into the muscles over the rib and into the spaces above and below the rib, was injected with cocaine and after waiting 20 or 30 seconds an incision was made down to the rib and through all structures. This was followed by considerable pain, in fact excessive pain, so it was necessary to try and recocainise the operative field. When five minutes had elapsed it was found that manipulation of the wound area produced no pain. Then a bone forceps was passed around the rib and the rib cut. This again was extremely painful to the patient, and mentally so to the operator. After removal of the piece of bone it was found that the pleural surface was very sensitive and could not be touched without further cocainisation.

After this was done a very small incision was made into the pleura and the pus slowly evacuated with the gloved finger acting as a valve, a careful watch being kept on the general condition of the patient. It was noticed that this patient was spared the usual hours of severe surgical shock and depression that always follow this operation when done under ether or chloroform. Instead, her convalescence started when she left the table.

In the analysis of that first case we find good physiological and good anatomical reasons why the procedure was not more successful in stopping pain. We know now that local anesthetics, to be of best use, must be infiltrated under pressure enough to measurably distend the tissues, and not simply injected into tissue spaces as we give an ordinary hypodermic. The sensory nerves of the chest and abdomen except at the lowest border come from the twelve pairs of costal nerves; these pass along the upper border of the rib next below and very quickly give off muscular branches that penetrate the muscles. The cutaneous branches perforate to the skin in front of the axillary line and close to the sternum. Shorter branches pass out through the muscles of the back near the spine. Also, we find them in the axillary line and in front of the subcostal muscles covering them. Long branches pass along on the posterior surface of the rib. These must be remembered and most carefully infiltrated if we would accomplish successful anesthesia.

Since the initial case this method has been used by the writer in ten or a dozen other cases with most satisfactory results. Every operation is done with the patient lying flat on the back, with the side projecting over the edge of the table. This is harder for the operator for the operation has to be done up hill, but there is no danger of drowning the patient by reason of a ruptured bronchus. A recent case, a child two and a half year's old, was operated at the children's hospital in this manner and when I say the child did not scream once through the operation you will believe that the procedure was successful in preventing pain. Two and a half years' old children, that have been sick for some time, do not hide their emotions.

The next class of patients in which this method seems particularly indicated is in any abdominal lesion where there is an accompanying stercoraceous vomiting. The writer has seen three patients when under chloroform drown in their own vomit, and this when the stomach had been supposedly emptied by a tube. From the comparison of recent results with local anesthesia as against general in strangulated hernias of 48 or more hours standing, we find that more of them recover when done with cocaine. In the very desperate cases the best results seem to follow opening the sac under cocaine, enlarging the ring, leaving the gangrenous gut in situ and opening it. If patients will not recover under this least of interference it is tolerably certain they will not recover after resection of the gangrenous area and closing of the hernia under chloroform. Later a closure of the artificial anus may be done safely.

Sometimes one meets with slowly growing very large appendiceal abscesses that have produced such a condition; in which case one hesitates to use a general anesthetic. In cases of this kind local anesthesia is just as satisfactory as in opening the chest, and the writer some months ago evacuated an abscess in this way and later under chloroform removed a large inflamed appendix.

Ordinarily we do not associate gall-bladder surgery with local anesthesia, but in getting ready to operate a patient with a long standing cholecystitis, a cholangitis edema of the extremities and ascites, it was considered advisable to try cocaine as the patient's pulse at the wrist was almost non-existant. It was found, however, that it was impossible to entirely anesthetise the parietal peritoneum as the ascites would dilute the cocaine swabs held against it, so the operation of opening and fixing a drainage tube in the gall-bladder was completed under a few whiffs of chloroform. This patient had practically no post-operative shock.

Now as to the method of employment. I believe that it makes much less difference what anesthetising solution you use than how you use it. To successfully anesthetise by cocaine a considerable area, either you must inject a strong solution such as 4% and wait five minutes for it to be thoroughly absorbed, or else if a weak solution is used it must be injected forcibly, distending the tissues so that they present the picture of a profound edema. In a case of a very old woman with a strangulated femoral hernia of over forty-eight hours' duration no cocaine was available when the patient was prepared for operation. As a substitute, a weak solution of morphine was prepared and this infiltrated into the tissues. She never made a protest until the peritoneum, which had not been properly injected, was nicked.

To try and satisfy my own mind as to the relative importance of the solution used, or the manner of using it, I had a patient prepared for a double herniotomy. With a large syringe and a small needle the skin was infiltrated with plain sterile warm water. Next the fatty tissue was blown up and then an incision carried to the external oblique. The patient said he didn't feel anything. By balooning up each layer of muscle and fascia with warm water and massaging it before it was cut, it was found that anesthesia was as complete as if a strong cocaine solution had been used.

In conclusion I would say that,

1. Certain cases of profound sepsis, notably empyema and localised abscess of the abdomen, are more safely attacked under local anesthesia.

2. The method of usage is more important in achieving good results than the agent used.

516 FRANKLIN STREET.

